# Therapeutic Effects of Plasmapheresis on Acute Exacerbations of Chronic Hepatitis B Infection

**DOI:** 10.7759/cureus.12779

**Published:** 2021-01-19

**Authors:** Yilmaz Bilgic, Sami Akbulut, Ayse Cengiz, Ahmet Sarici, Yasir Cagin, Murat Harputluoglu

**Affiliations:** 1 Gastroenterology and Hepatology, Inonu University, Malatya, TUR; 2 Surgery/Liver Transplantation, Surgery and Liver Transplant Institute, Inonu University Faculty of Medicine, Malatya, TUR; 3 Internal Medicine, Inonu Univeristy, Malatya, TUR; 4 Hematology, Inonu University, Malatya, TUR

**Keywords:** chronic hepatitis b, execarbation, plasmapheresis, liver transplantation

## Abstract

Objective

In this study, we aimed to demonstrate the effectiveness of plasmapheresis therapy in patients with acute exacerbation of chronic Hepatitis B (CHB) infection.

Methods

We selected 48 patients with acute exacerbation of CHB infection who were treated by plasmapheresis in our intensive care unit between 2009 and 2016. The patients' demographic characteristics and biochemical and hematological parameters, which were recorded before and after plasmapheresis, were assessed, and the effect of plasmapheresis on the course of patients' treatment was examined. The patients were also divided into three groups according to their clinical course (discharged: 24; transplanted: six; exitus: eight). The patients were further divided into four groups and compared based on the underlying causes that led to the exacerbation (spontaneous exacerbation: 25; caused by immunosuppressive drugs: nine; hepatotoxic drugs: six; other agents: eight).

Results

We observed significant improvements in terms of international normalized ratio (INR), aspartate aminotransferase (AST), alanine aminotransferase (ALT), gamma-glutamyl transferase (GGT), lactate dehydrogenase (LDH), total bilirubin, direct bilirubin, blood urea nitrogen (BUN), ammonia, and the Model for End-Stage Liver Disease (MELD) score after plasmapheresis therapy. However, there was no significant improvement in hemoglobin (Hb), white blood cell (WBC) count, platelets, albumin, and lactate values. Also, INR, ALP, and ALT values were found to be significantly correlated with transplants and exitus in patients.

Conclusion

Plasmapheresis therapy is a reliable treatment method that provides clinical recovery and improvement in laboratory parameters in patients with exacerbation of CHB infection.

## Introduction

Hepatitis B virus (HBV) is a partly double-stranded, hepatotropic virus that belongs to the orthohepadnavirus genome, and chronic hepatitis B (CHB) infection is a serious health problem globally [1]. CHB infection is a dynamic process and the progression of liver damage in patients depends on the age at which the infection occurs, the presence of viral replication, and the interaction between the virus and the host [2]. HBV-associated acute hepatitis may either be a true episode of acute hepatitis B infection or an acute exacerbation of previously unknown CHB infection. Acute exacerbation of hepatitis B infection is not common, and its cumulative incidence is estimated to be 10-30% per year.

Severe acute exacerbations may develop during the period of inactive hepatitis, although they mostly occur in the immunoclearance phase [3]. Acute hepatic exacerbations can occur due to a spontaneous exacerbation of immune response to HBV, exposure to immunosuppressive agents, or immunodeficiency; it can also result from a re-immune response to HBV following an interruption in the immunosuppressive treatment, alteration of the virus structure, the addition of other viruses such as the hepatitis C virus (HCV), hepatitis D virus (HDV), and human immunodeficiency virus (HIV), and alcohol or drug exposure that causes liver damage [4]. Acute exacerbations may be mild or severe with hepatic decompensation [5].

Extracorporeal liver support systems can be basically divided into three main categories: biological, non-biological (artificial), and bio-artificial (hybrid techniques) [6]. Non-biological liver support systems are further classified into the molecular adsorbent recirculating system, single-pass albumin dialysis, fractionated plasma separation and adsorption, and therapeutic plasma exchange (plasmapheresis) [7]. Plasmapheresis is one of the non-biological liver support systems, and it involves the process of removing the patient's plasma and replacing it with replacement fluids [8,9]. The purpose of therapeutic plasma exchange is to reduce the plasma components, which are known to be associated with the etiopathogenesis of a variety of diseases, thereby reducing the pathological damage to the organism or reversing this damage to some extent [10,11]. Examples of plasma proteins, which may be responsible for disease pathogenesis and are thought to be harmful to the patient, include monoclonal proteins, cryoglobulins, immunocomplexes, lipoproteins, autoantibodies, alloantibodies, and toxins [12].

Plasmapheresis involves the removal of plasmid from the body and replacing it with albumin or plasma [13,14]. It is a nonselective method that can remove harmful and toxic substances from blood circulation [15,16]. In general, plasma exchange is recommended three times a week for a few weeks if circulating immune complexes, myeloma proteins, and immunoglobulin G (IgG) and IgM type antibodies are intended to be removed. The plasma exchange time should be adjusted and gradually reduced by taking into account the laboratory data and the evolving clinical situation [17,18]. The primary aim of this study was to demonstrate the effectiveness of plasmapheresis therapy in patients with acute exacerbation of CHB infection.

## Materials and methods

For this study, we selected patients with acute exacerbation of CHB infection who were treated by plasmapheresis in the Department of Gastroenterohepatology, Inonu University Faculty of Medicine Intensive Care Unit between 2009 and 2016. Patients' demographic characteristics and biochemical parameters, which were recorded before and after plasmapheresis, were retrospectively evaluated to assess the effectiveness of plasmapheresis. Chronic HBV infection was defined as persistent seropositivity of hepatitis B surface antigen (HBsAg) during the entire follow-up period [19]. Acute exacerbation of CHB infection was defined as intermittent elevations of aminotransferase activity to more than 10 times the upper limit of normal and more than twice the baseline value [20]. As a result of the file scanning based on the above-mentioned criteria, a total of 62 patients were initially considered, but 14 patients were excluded from the study due to a lack of clarity regarding their demographic characteristics and biochemical data. Thus, a total of 48 patients were included in this study. Patients with conditions such as alcoholism, ischemia, pregnancy, and those with non-B hepatitis that caused similar biochemical profiles were excluded. Patients with other hepatotropic viruses were also excluded. Biochemical parameters of the patients were compared based on the following variables: timing of biochemical analysis (baseline vs. after 48 hours vs. last available), final status (exitus vs. transplanted vs. recovered), factors affecting exacerbation (spontaneous vs. hepatotoxic drugs vs. immunosuppressive drugs vs. other agents). The following clinical and biochemical parameters were analyzed: age in years, body mass index (BMI), hemoglobin (Hb), white blood cell (WBC), thrombocytes, aspartate aminotransferase (AST), alanine aminotransferase (ALT), lactate dehydrogenase (LDH), alkaline phosphatase (ALP), gamma-glutamyl transferase (GGT), total bilirubin, direct bilirubin, international normalized ratio (INR), albumin, ammonia, lactate, and the Model for End-Stage Liver Disease (MELD) score.

## Results

A total of 48 patients who were aged between 18 and 70 years were included in this study; there were 34 male and 14 female patients. The median age of male and female patients was 47.7 years (range: 31-70 years) and 38.4 years (range: 18-49 years), respectively. The mean BMI of the patients was 22.8 ± 4.7 kg/m^2^, and the mean duration of HBV infection was 42.72 ± 53.8 months. The number of patients with known HBV history in the family was 21 (43.7%), and the number of patients with or without a history of HBV in the family was 27 (56.25%). There were six patients (12.5%) with a history of alcohol use and 19 (39.6%) patients with a smoking history. The number of patients with a history of hepatotoxic drug and herbal medicine use was two (1.6%), and patients with immunosuppressive drug use constituted 6.4% of the cohort. However, the use of these drugs and herbal medicine had occurred long before they had led to the exacerbation, and had been abandoned.

Regarding the causes of acute exacerbations in patients, there were 25 (52.1%) patients in the spontaneous group; while nine (18.7%) patients had acquired it due to immunosuppressive drugs, it had been caused by hepatotoxic drugs in six (12.5%) patients and the use of other agents in eight patients (16.7%) [anti-tumor necrosis factor (anti-TNF) drugs: two; chemotherapy: four; rituximab: two]. The demographic data of the patients and the causes of exacerbation are presented in Table 1. After the plasmapheresis treatment, significant amelioration was observed among patients in terms of INR (p=0.001), AST (p=0.027), ALT (p=0.007), ALP (p<0.001), GGT (p=0.001), LDH (p=0.001), total bilirubin (p=0.001), direct bilirubin (p=0.008), ammonia (p=0.05), and the MELD score (p=0.003). However, no significant changes were observed in Hb, WBC, thrombocytes, albumin, and lactate levels (Table 2).

**Table 1:** Characteristics of patients with chronic hepatitis B infection exacerbation CHBI: chronic hepatitis B infection; TNF-alpha: tumor necrosis factor-alpha

Parameters	N	%
Gender		
Male	34	70.1
Female	14	29.9
Factors causing the exacerbation of CHBI		
Group I (spontaneous)	25	52.1
Group II (hepatotoxic drug use)	6	12.5
Group III (immunosuppressive drug use)	9	18.7
While using immunosuppressive drugs	4	
After using immunosuppressive drugs	5	
Group IV (other drug use)	8	
While using chemotherapeutics	4	
While using anti-TNF-alpha	2	
Rituximab	2	16.7

**Table 2:** Laboratory effects of plasmapheresis on patients with chronic hepatitis B infection exacerbation Hb: hemoglobin; WBC: white blood cells; INR: international normalized ratio; AST: aspartate aminotransferase; ALT: alanine aminotransferase; LDH: lactate dehydrogenase; ALP: alkaline phosphatase; GGT: gamma-glutamyl transferase; MELD: Model for End-Stage Liver Disease

Parameters	Baseline, median (min-max)	After 48 hours, median (min-max)	Last available, median (min-max)	P-value
Hb, g/dL	10.8 (6.7-15.9)	10.2 (6.4-14.7)	10.5 (6.9-15.2)	0.073
WBC, x 10E^3^/uL	9.1 (2.4-28.1)	9.6 (2.6-28.5)	9.4 (1.6-29.1)	0.060
Thrombocytes, x 10^9^/L	112 (43-354)	106 (41-311)	104 (27-298)	0.820
INR	2.6 (1-4.3)	1.1 (0.9-3.3)	1.1 (1-3.5)	<0.001
AST, IU/L	372 (54-6,404)	272 (38-5,624)	231 (32-3,151)	0.027
ALT, IU/L	449 (75-5,765)	384 (68-4,032)	283 (27-2,543)	0.007
ALP, IU/L	172 (30-732)	152 (34-528)	139 (11-352)	<0.001
GGT, IU/L	197 (38-597)	149 (32-502)	152 (33-484)	0.001
LDH, IU/L	426 (119-1,007)	321 (86-752)	364 (145-1,200)	0.001
Total bilirubin, mg/dL	16.4 (14.5-37.9)	12 (9.8-24.7)	13 (3.8-30.3)	0.001
Direct bilirubin, mg/dL	10.2 (2.1-25.9)	8.3 (1.6-16.2)	7.9 (1.5-21.6)	0.008
Albumin, g/dL	2.85 (2.1-3.8)	2.9 (2.4-3.8)	2.86 (1.8-3.9)	0.550
Ammonia, mcg/dL	135 (58-465)	27.4 (47-387)	30.4 (47-227)	0.050
Lactate, mmol/l	21.3 (8.3-120)	21.2 (8.1-115)	21.5 (5.4-71.6)	0.416
MELD score	15 (10-32)	12 (9-26)	10 (8-21)	0.003

Patients were divided into three groups in terms of final status: those who were deceased (n=8), those who were transplanted (n=16), and those who recovered (n=24). There were statistically significant differences between deceased and recovered groups in terms of creatinine (p=0.018), Hb (p<0.001), thrombocytes (p=0.001), INR (p=0.001), GGT (p=0.015), AST (p=0.018), ALT (p=0.014), lactate (p<0.001), and the MELD score (p=0.01). Also, there were statistically significant differences between transplanted and deceased groups in terms of Hb (p=0.018), thrombocytes (p=0.006), INR (p=0.002), ALP (p=0.002), ALT (p=0.007), lactate (p=0.001), and ammonia levels (p=0.013). Morever, there were statistically significant differences between the recovered and and transplanted groups in creatinine (p=0.016), ALP (p=0.001), GGT (p=0.001), AST (p=0.036), direct bilirubin (p=0.043), and the MELD score (p=0.01) (Table 3).

**Table 3:** Comparison of the biochemical analysis of deceased, transplanted, and recovered patients who received plasmapheresis *Significant difference between recovered and deceased; ^β^significant difference between transplanted and deceased; ^+^significant difference between recovered and transplanted Hb: hemoglobin; WBC: white blood cells; INR: international normalized ratio; AST: aspartate aminotransferase; ALT: alanine aminotransferase; LDH: lactate dehydrogenase; ALP: alkaline phosphatase; GGT: gamma-glutamyl transferase; MELD: Model for End-Stage Liver Disease

Parameters	Deceased (n=8), median (min-max)	Transplanted (n=16), median (min-max)	Recovered (n=24), median (min-max)	P-value
Age, years	48 (32-70)	45 (29-58)	37 (18-54)	0.146
Creatinine, mg/dl	1.4 (0.5-2.1)	1.2 (0.7-2.4)	0.5 (0.3-1.0)	0.018*/0.016^+^
Hb, g/dL	9.5 (6.7-12.7)	10.4 (7.9-12.9)	11.1 (10.1-15.9)	<0.001*/0.018^β^
WBC, x 10E^3^/uL	9.4 (3.2-28.5)	9.7 (1.6-21.4)	9.2 (2.4-22.7)	0.189
Thrombocytes, x 10^9^/L	125 (43-324)	144 (87-226)	152 (75-354)	0.001*/0.006^β^
INR	2.6 (1.7-4.3)	2.4 (1.8-3.7)	1.84 (1-3.2)	0.001*/0.002^β^
AST, IU/L	586 (116-6,404)	485 (154-5,418)	341 (54-4,267)	0.018*/0.036^+^
ALT, IU/L	486 (75-5,765)	470 (91-5,456)	428 (89-5,216)	0.007^β^/0.014*
ALP, IU/L	224 (30-732)	251 (56-554)	188 (77-356)	0.001^+^/0.002^β^
GGT, IU/L	224 (44-514)	251 (87-597)	203 (72-472)	0.001^+^/0.015*
LDH, IU/L	544 (256-1,007)	579 (287-946)	412 (119-786)	0.960
Total bilirubin, mg/dL	18.4 (14.5-37.9)	15.4 (12.7-28.7)	12.2 (14.8-24.7)	0.590
Direct bilirubin, mg/dL	14.2 (10.1-25.4)	10.1 (8.8-25.9)	8.7 (2.1-18.9)	0.043^+^
Albumin, g/dL	2.3 (2-3.2)	2.4 (2.2-3.3)	2.9 (2.6-3.8)	0.850
Ammonia, mcg/dL	138 (58-384)	132 (72-455)	132 (63-465)	0.013^β^
Lactate, mmol/l	23.5 (14.4-120)	22.8 (15-112)	20.8 (8.3-110)	<0.001*/0.01^+^
MELD score	18 (16-30)	19 (16-32)	14 (10-21)	0.01*/0.01^+^

Patients were divided into four groups based on the factors causing the exacerbation of CHBI. Statistical analysis was performed to determine whether plasmapheresis was effective among these groups (spontaneous exacerbation and other drug groups), and there was a statistically significant difference in terms of total bilirubin (p=0.04) and direct bilirubin (p=0.03) values (Table 4).

**Table 4:** Comparison of the efficacy of the plasmapheresis between groups created based on underlying causes INR: international normalized ratio; AST: aspartate aminotransferase; LDH: lactate dehydrogenase

Parameters	Spontaneous, median (min-max)	Hepatotoxic drugs, median (min-max)	Immunosuppressive agents, median (min-max)	Other drugs, median (min-max)	P-value
INR	2.1 (1.3-3)	3.2 (1.2-4.3)	2.9 (1.8-3.2)	3.2 (1-3.7)	0.320
AST, IU/L	498 (116-771)	1,593 (228-6,404)	1,023 (245-1,412)	544 (229-654)	0.570
LDH, IU/L	486 (354-624)	863 (119-1,007)	616 (321-745)	631 (358-958)	0.280
Total bilirubin, mg/dL	18 (12.7-25.8)	18.4 (12.4-22.1)	19 (12.4-24.7)	29.6 (24.7-37.9)	0.040
Direct bilirubin, mg/dL	12 (2.1-18.9)	12 (3.1-16.5)	12.5 (3.2-20.4)	20.3 (18.7-25.9)	0.030
Albumin, g/dL	2.86 (2.6-3.8)	2.85 (2.4-3.5)	2.73 (2.6-2.9)	2.36 (2.0-2.7)	0.080
Ammonia, mcg/dL	120 (62-157)	240 (178-465)	183 (94-214)	118 (58-144)	0.620

## Discussion

There is a dynamic process in CHB infection. In other words, while it may continue to be asymptomatic in patients, it can also be associated with a course that leads to fulminant hepatitis. Viral infections, interstitial toxic drugs used for other reasons, the age at which the patient was exposed to the virus, changes in the genetic structure of the virus, and the use of immunosuppressive drugs or the development of another immunosuppressive condition can affect the duration of the virus infection. Cytokines occur as a result of various stimuli in CHB exacerbations. The removal of these cytokines in the dynamic process of CHB exacerbation process can reduce cytokine damage to the liver and contribute to the development of balance in the body. Various treatments are performed in this patient group. One of these treatment methods is called plasmapheresis. Plasmapheresis is classified as indication category III by the American Association of Blood Banks (AABB) [21,22]. In this study, we aimed to demonstrate the effectiveness of plasmapheresis on laboratory and clinical parameters in patients with acute HBV exacerbation.

A moderate increase in hyperbilirubinemia, ALP, and transaminase levels may be observed in this condition. High liver enzymes are a component of the hemolysis, elevated liver enzymes, low platelet count (HELLP) syndrome. Elevated liver enzymes are believed to be caused by the slowing of the secondary blood flow to fibrin deposits in the hepatic sinusoids [23]. Both experimental and clinical studies have shown that there is a significant decrease thanks to plasmapheresis in TNF-alpha, interleukin-6 (IL-6), and endotoxins levels, which play a role in the pathogenesis of systemic inflammatory response syndrome (SIRS) and sepsis [24,25]. AST and GGT are liver enzymes showing cell destruction, and in our study group, plasmapheresis significantly improved these two laboratory values. However, as shown in many studies, the improvement in AST and GGT values did not correlate with improvements in liver histology [26].

In a study by Tsai et al. [27], high AST and bilirubin levels, low albumin and platelet levels, and prolonged prothrombin time (PT) were reported as prognostic factors of acute exacerbations of CHB. A study examining the effects of plasmapheresis and hemodiafiltration separately and in combination with cytokines has revealed that a single plasmapheresis treatment significantly managed to reduced IL-6 and bilirubin levels but no significant changes in TNF-alpha and IL-8 levels were observed [28].

In a study of acute liver failure patients, it was shown that plasmapheresis provides a significant decrease in ammonia levels [29]. Another study has reported that in patients with newborn hyperbilirubinemia, bilirubin levels were significantly reduced by plasmapheresis treatment [30]. In our study, after the plasmapheresis treatment, there was significant amelioration in INR, AST, ALT, ALP, GGT, LDH, total bilirubin, direct bilirubin, ammonia, and the MELD score. However, no significant changes were observed in Hb, WBC, platelets, albumin, and lactate levels.

We believe that the therapeutic effect of plasmapheresis is more effective in acute exacerbation of CHB that occurs as spontaneous events and in immunosuppressive patients compared to other exacerbations. We also hold the view that plasmapheresis may improve laboratory values and clinical course in acute exacerbation of hepatitis B and may serve as a bridge, especially in patients waiting for liver transplantation. Eight of the 48 patients in our study had died, 16 of them had been transplanted, and 24 of them were discharged. It is also necessary to consider the factors accompanying CHB that cause mortality in patients who are followed up in the intensive care. Immunosuppressant use, anti-TNF use, and chemotherapeutic treatments cause acute exacerbations in CHB, as well as suppressing the immune system and making the patient vulnerable to other infections. While acute exacerbations of CHB already have a picture similar to that of SIRS, hospital infections added to it accelerate this process and lead the patient to a process that results in septic shock and thereby increase mortality. Therefore, these factors are also likely to have constituted a basis for the illness among the patients who had died in our cohort. There are no studies in the literature related to this subject; hence, additional studies are warranted so that more light is shed on this topic. Similarly, additional factors such as diabetes mellitus, metabolic factors such as obesity, persistent activation of liver disease, frequent exacerbations, high geographic endemicity, the genotype of the virus, and pre-core or core promoter variants also affect the natural course of CHB and alter the response to plasmapheresis. Further studies are therefore required, including those involving more cases and factors that affect the natural course of CHB.

Our patients were divided into three groups: those who died, transferred, and recovered, respectively. There was a statistically significant difference between the excluded group and the recovered group in terms of creatinine, Hb, thrombocytes, INR, GGT, AST, ALT, lactate, and the MELD score, and, additionally, there was a statistically significant difference between the transplanted group and the excluded group in terms of Hb, thrombocyte, INR, ALP, ALT, lactate, and ammonia; moreover, there was a statistically significant difference between the recovery group and the transplanted group pertaining to creatinine, ALP, GGT, AST, direct bilirubin, and the MELD score.

Based on our findings, changes in biochemical parameters contribute to mortality in CHB exacerbations. Biochemical, hematologic, and MELD score changes with plasmapheresis may have a positive effect on survival in these patient groups. In our study, patients were further divided into four groups in order to investigate whether there was any difference in the effectiveness of plasmapheresis based on the different etiologic agents that had caused the exacerbation of the CHB infection. The statistical analysis that determined the efficacy of plasmapheresis among these groups showed a significant association between the spontaneous exacerbation group and the other-drug groups only in total bilirubin and direct bilirubin. That is, in the spontaneous CHB exacerbation group, total and direct bilirubin values decreased with plasmapheresis treatment compared to the other groups. This has led us to believe that this treatment method may be more beneficial in this group of patients. However, further randomized prospective controlled studies involving larger groups of patients are needed to arrive at a comprehensive and definitive understanding of this subject.

## Conclusions

In conclusion, there are no other studies in the literature similar to our current study, and the results of this study have shown that plasmapheresis is an effective treatment modality for patients with acute exacerbation of CHB infection.
